# The effect of psychoactive bacteria, *Bifidobacterium longum* Rosell®-175 and *Lactobacillus rhamnosus* JB-1, on brain proteome profiles in mice

**DOI:** 10.1007/s00213-023-06519-z

**Published:** 2023-12-29

**Authors:** Łukasz S. Jarosz, Katarzyna Socała, Katarzyna Michalak, Adrian Wiater, Artur Ciszewski, Małgorzata Majewska, Agnieszka Marek, Zbigniew Grądzki, Piotr Wlaź

**Affiliations:** 1 Department of Epizootiology and Clinic of Infectious Diseases, Faculty of Veterinary Medicine, University of Life Sciences in Lublin, Głęboka 30, 20-612 Lublin, Poland; 2 Department of Animal Physiology and Pharmacology, Institute of Biological Sciences, Faculty of Biology and Biotechnology, Maria Curie–Skłodowska University, Akademicka 19, 20–033 Lublin, Poland; 3 Department of Industrial and Environmental Microbiology, Institute of Biological Sciences, Faculty of Biology and Biotechnology, Maria Curie–Skłodowska University, Akademicka 19, 20–033 Lublin, Poland; 4 Department of Preventive Veterinary and Avian Diseases, Faculty of Veterinary Medicine, University of Life Sciences in Lublin, Głęboka 30, 20-612 Lublin, Poland

**Keywords:** Mouse, Gut-brain axis, Psychoactive bacteria, Hippocampus, Proteomic profile, MALDI-TOF MS

## Abstract

**Rationale:**

The gut microbiota may play an important role in the development and functioning of the mammalian central nervous system. The assumption of the experiment was to prove that the use of probiotic bacterial strains in the diet of mice modifies the expression of brain proteins involved in metabolic and immunological processes.

**Objectives and results:**

Albino Swiss mice were administered with *Bifidobacterium longum* Rosell®-175 or *Lactobacillus rhamnosus* JB-1 every 24 h for 28 days. Protein maps were prepared from hippocampal homogenates of euthanized mice. Selected proteins that were statistically significant were purified and concentrated and identified using MALDI-TOF mass spectrometry. Among the analysed samples, 13 proteins were identified. The mean volumes of calcyon, secreted frizzled-associated protein 3, and catalase in the hippocampus of mice from both experimental groups were statistically significantly higher than in the control group. In mice supplemented with *Lactobacillus rhamnosus* JB-1, a lower mean volume of fragrance binding protein 2, shadow of prion protein, and glycine receptor α4 subunit was observed compared to the control.

**Conclusion:**

The psychobiotics *Bifidobacterium longum* Rosell®-175 and *Lactobacillus rhamnosus* JB-1enhances expression of proteins involved in the activation and maturation of nerve cells, as well as myelination and homeostatic regulation of neurogenesis in mice. The tested psychobiotics cause a decrease in the expression of proteins associated with CNS development and in synaptic transmission, thereby reducing the capacity for communication between nerve cells. The results of the study indicate that psychobiotic bacteria can be used in auxiliary treatment of neurological disorders.

## Introduction

Widespread chemical treatment of infectious and non-infectious diseases, poor diet, and numerous stress factors are attributes of the contemporary fast pace of human life (Cohen et al. 2015), as well as industrial livestock farming focused on attaining the highest possible production (Graham et al. 2008; Manyi-Loh et al. 2018; Budreviciute et al. 2020). A common phenomenon in these conditions, observed in both humans and animals, is functional disorders of the gastrointestinal tract (GIT), whose functioning largely depends on the intestinal microbiota (Carding et al. 2015). Often underlying gastrointestinal dysfunction are changes in the intestinal microbiome (dysbiosis), especially disturbances in the quantitative and qualitative composition of microbes colonizing the gastrointestinal mucosa (Sekirov et al. 2010; Gomaa 2020). These microbes, owing to their interactions and action on the host, can take part in numerous physiological processes, including digestion, stimulation of local and systemic defence mechanisms, maintenance of homeostasis, and other phenomena determining the normal development of the body (Belkaid and Hand 2014; Rooks and Garrett 2016). Disturbances of the intestinal microbiome have long been known to accompany many disease states, including diabetes and obesity, as well as diseases with inflammation (Musso et al. 2010). Recently published results have shown that the intestinal microbiota also plays an important role in the development and functioning of the central nervous system (CNS) and can influence cognitive functions by acting on metabolic, neuroendocrine, and immune pathways (Carabotti et al. 2015; Zhu et al. 2017; Cerdó et al. 2020). These studies are evidence of two-way communication between the brain and the intestinal microbiota, known as the microbiota–gut–brain axis (Carabotti et al. 2015; Appleton 2018; Chakrabarti et al. 2022). The effects of the intestinal microbiome on the nervous system are usually multifaceted and include its effect on the sensory nerve fibres, including the vagus nerve, which mediate transmission of information to the CNS and decrease the perception of visceral pain (Wang and Kasper 2014; Moloney et al. 2016; Mitrea et al. 2022). Interestingly, disturbances in the intestinal microbiome can also be associated with mood disorders, depression, and anxiety, which has been demonstrated in human subjects with irritable bowel syndrome (Cryan and O’Mahony 2011; Kumar et al. 2023). One of the strategies currently promoted for restoring the microbiological balance of the intestines is diet supplementation with probiotics containing selected strains of various microbial species (Hemarajata and Versalovic 2013). The beneficial effect of probiotic microbes on the body is manifested as regulation of intestinal function, stabilization and maintenance of the balance between pathogenic and saprophytic microbes and stimulation of enterocyte development. Probiotics take also part in the regulation of gastrointestinal motility, increase digestion and absorption of proteins, carbohydrates and fats, and produce biologically active compounds such as enzymes and vitamins (Wang et al. 2021). Probiotic bacteria also stimulate local (GALT, gut-associated lymphoid tissue) and systemic host immune mechanisms, expressed as modulation of T and B cell functions and stimulation of immunocompetent cells to produce cytokines, which regulate the systemic and local immune response (Hardy et al. 2013; Wang et al. 2021). Another positive effect of probiotics is inhibition of inflammation of the intestinal mucosa through stabilization of the environment of bacteria and proliferation and cytokine activation of NK cells (Cristofori et al. 2021). The multifaceted potential effects of probiotics on the body, especially the hypothesis regarding their interactions with the CNS mediated by the microbiota–gut–brain axis, have prompted the implementation of new methods of prevention and treatment of emotional and mental disorders in people, involving the use of psychobiotics as diet supplements (Dinan 2013; Sarkar et al. 2016; Mörkl et al. 2020; Berding et al. 2023).

The concept of psychobiotics refers to a large group of probiotics containing bacterial strains which have no pro-inflammatory lipopolysaccharide chains and do not induce an acute inflammatory response in the intestines or prebiotics which during fermentation of food in the intestines induce changes in the composition or activity of the bacteria constituting the microbiome. Both groups of microbes, when ingested in appropriate quantities, exert positive psychiatric effects in psychopathology (Bermúdez-Humarán et al. 2019; Del Toro-Barbosa et al. 2020; Oroojzadeh et al. 2022). The mechanism of action of psychobiotics is not fully known but is assumed to rely on stimulation of the intestinal nervous system, the immune and endocrine systems, and metabolic processes. The use of psychobiotics in humans has also been shown to influence psychophysiological markers of depression and anxiety, as well as inflammation (Sarkar et al. 2016; Gualtieri et al. 2020). Underlying this effect is the influence of the hypothalamic–pituitary–adrenal axis (HPA) on the stress response, a reduction in systemic inflammation, direct effects on the immune system, and also synthesis of neurotransmitters, proteins, and short-chain fatty acids (Dinan 2013; Sarkar et al. 2016; Cheng et al. 2019; Del Toro-Barbosa et al. 2020; Zielińska et al. 2022).

The bacterial strains most often used to produce psychobiotics include *Bifidobacterium longum* Rosell®-175 and *Lactobacillus rhamnosus* JB-1 (Allen et al. 2016; Kelly et al. 2017; Forssten et al. 2022)*.* Bravo et al. (2011) demonstrated that the use of *Lactobacillus rhamnosus* JB-1 in mice reduced the level of corticosterone in the blood and mitigated depressive and anxious behaviours by reducing expression of the GABAB1β receptor in the hippocampus and amygdala. Diet supplementation with *Lactobacillus rhamnosus* JB-1 also affected metabolic processes in the brain of mice with stress-associated anxiety disorders, resulting in a reduction in stress severity measured using behavioural tests (Xu et al. 2022). In addition, diet supplementation with this probiotic decreased the response of the HPA axis to stress and expression of specific GABA receptors in individual regions of the brain (Kochalska et al. 2020; Chudzik et al. 2022). Bharwani et al. (2017) and Marin et al. (2017) used a model of chronic psychosocial stress to show that *L. rhamnosus* JB-1 alleviates anxiety-like behaviours, reduces deficiencies in social interactions, and exerts an immunoregulatory effect. Recent research indicates that the development of psychological disorders such as depression is linked to an increase in inflammation and activation of pro-inflammatory cytokines such as interleukin-6 (IL-6), IL-1β, and tumour necrosis factor α (TNF-α), which indirectly affect the composition of the intestinal microbiome (Lotrich 2015; Maeng and Hong 2019). The use of *Lactobacillus rhamnosus* JB-1 in mice, on the other hand, reduced the concentration of pro-inflammatory cytokines and mitigated anxiety disorders (Mindus et al. 2021; Chudzik et al. 2022).

Similar effects have been shown for *Bifidobacterium longum* Rosell®-175. The use of this strain in mice in combination with *Lactobacillus helveticus* R0052 influenced the HPA axis in a state of chronic stress in mice, alleviating its effects (Ait-Belgnaoui et al. 2014). The combined use of *Lactobacillus helveticus* R0052 and *Bifidobacterium longum* R0175 in rats and humans reduced cortisol concentrations in the urine, which confirms the anti-anxiety and anti-stress effects of these microbes (Messaoudi et al. 2011).

The multifaceted effects of psychobiotics containing *Bifidobacterium longum* Rosell®-175 and *Lactobacillus rhamnosus* JB-1 are linked to stimulation of organ metabolism and the immune and endocrine systems, and also result from activation of specific genes located in the brain, endocrine glands or other tissues (Poluektova et al. 2021). The mechanisms causing metabolic changes in the brain and resulting in a change in the protein profile as a consequence of diet supplementation with psychobiotics containing *Lactobacillus rhamnosus* JB-1 and *Bifidobacterium longum* Rosell®-175 are as yet unknown. We hypothesized that supplementation of the diet of mice with *Lactobacillus rhamnosus* JB-1 and *Bifidobacterium longum* Rosell®-175 modifies the expression of brain proteins involved in metabolic and immune processes. The aim of the study was to identify proteins synthesized in the brain of mice fed a diet supplemented with *Lactobacillus rhamnosus* JB-1 or *Bifidobacterium longum* Rosell®-175, to determine the differences in the expression of these proteins in comparison with the control group. The results should provide the first scientific evidence of differentiation of the proteomic profile of the brain as a result of diet supplementation with psychobiotics.

## Materials and methods

### Animals

Thirty male albino Swiss mice were used in this study. The animals were purchased from a licensed breeder (Kołacz, Laboratory Animals Breeding, Warsaw, Poland) at age of 5–6 weeks and were kept under controlled environmental conditions (21–24 °C; 45–65% humidity; 12-h light/dark cycle; light on at 6:00 a.m.) with free access to tap water and standard laboratory chow (Agropol S.J., Motycz, Poland). They were housed in groups in standard transparent cages (37 cm × 21 cm × 14 cm) and habituated for 7 days before starting the treatment. Housing and experimental procedures were performed in accordance with the EU council directive 2010/63/EU and Polish legislation concerning animal experimentation. The experimental protocol was approved by the Local Ethical Committee in Lublin, Poland (license no 65/2022).

### Bacterial preparation

*Lactobacillus rhamnosus* JB-1 (LR-JB1™) was gifted from Prof. Greg J. Stanisz. *Bifidobacterium longum* Rosell®-175 was obtained courtesy of SANPROBI Sp. z o.o., Sp. k. (Szczecin, Poland). Bacterial strains were stored as a frozen stock at − 80 °C in Man-Rogosa-Sharpe liquid medium (MRS broth; Difco Laboratories, Detroit, USA) containing 20% glycerol. From frozen stocks, bacteria were sub-cultured (overnight, in anaerobic conditions, 37 °C) in the MRS medium supplemented with 0.05% l-cysteine-HCl. The overnight cultures were transferred to fresh MRS broth (4.5 L) and again incubated at 37 °C for 48 h in anaerobic conditions (under mineral oil). After 2 days, cells were harvested from the growth medium by centrifugation at 9000 rpm for 20 min. The pellets were washed three times with sterile PBS buffer and re-suspended in sterile PBS (450 mL). The turbidity of bacterial suspension was compared to the McFarlands scale and its dilution was made in sterile PBS buffer to 1 × 10^10^ cfu/mL (2 × 10^9^ cfu/0.2 mL). The final bacterial suspensions were bottled into 50-mL Falcon tubes and stored at − 20 °C until the mice were fed.

### Treatment

Mice were divided into three groups (*n* = 10) and administered with: (Group I) 200 µL of PBS (control group), (Group II) 2 × 10^9^ cfu of *Bifidobacterium longum* Rosell®-175 in 200 µl PBS, and (Group III) 2 × 10^9^ cfu of *Lactobacillus rhamnosus* JB-1 in 200 µl PBS of by oral gavage every 24 h for a total of 28 days. See Fig. 9.

### Tissue collection

Two hours after the last treatment, the animals were sacrificed and the brains were rapidly dissected out and washed out in ice-sold saline (87 mM NaCl, 2.5 mM KCl, 1.25 mM NaH_2_PO_4_, 25 mM NaHCO_3_, 0.5 mM CaCl_2_, 7 mM MgSO_4_, 25 mM glucose, 75 mM sucrose, pH 7.4). Next the brain regions, hippocampus, from each animal were isolated and stored at − 80 °C until proteomic analysis according to the methods described by Wiśniewski and Gaugaz (2015) and Haas-Neill et al. (2022).

### Protein extraction

Hippocampus was cut into small pieces, washed in 0.9% NaCl, and homogenized (T10 basic IKA, Germany) in TRIS–HCl (1.5 M in water, pH 8.8). The samples were subsequently purified, desalted, and concentrated using Amicon Ultra-0.5 3 kDa centrifugal filter units (Merck KGaA, Darmstadt, Germany). Next, 180 µg of protein pellets were obtained using a precipitation kit (Ready-Prep™ 2-D Cleanup Kit, Bio-Rad, Warsaw, Poland) and dissolved in rehydration buffer (Bio-Rad, Warsaw, Poland). Protein mixtures were dropped onto a rehydration plate and covered with 17-cm immobilized pH gradient (IPG) linear strips for isoelectric focusing (ReadyStrip IPG, pH 3–10, Bio-Rad, Warsaw, Poland). The strips were overlaid with mineral oil to prevent them from drying out (Mineral oil, Bio-Rad, Warsaw, Poland). The strips were left to rehydrate for 12 h. Next, strips with soaked proteins were put in an IEF-100 Hoefer apparatus (Hoefer IEF100, Hoefer, Inc., Holliston, MA, USA) for electrophoretic isofocusing under the following conditions: 250 V/30 min; 10,000 V/3 h; 60 kV/h, with a current limit of 50 µA/strip. Before the second dimension, strips with focused proteins were equilibrated in 1,4-dithiothreitol and iodoacetamide solutions. Then, the strips were transferred onto 12.5% polyacrylamide gels and subjected to the second dimension of electrophoresis under 600 V/30 mA/100 W in an electrophoretic chamber (PROTEAN® II xi, Bio-Rad, Warsaw, Poland). After separation was completed, the gels were subjected to a standard silver-staining process in the presence of formaldehyde. Next, the gels were digitalized by scanning (Image Scanner III, GE Healthcare, Warsaw, Poland) and processed with Delta2D software (version 4.7, DECODON, Greifswald, Germany). Gel images were warped, which means that spots of the same protein had the same position across all gels in the experiment, and were fused. A fused image can be defined as a protein map containing every protein spot obtained during the experiment. Expression ratios were generated after the assumptions were checked based on the Shapiro–Wilk test (*α* = 0.05) and statistics were calculated for normalized volumes by one-way ANOVA (*P* value ≤ 0.05) and a post hoc Tukey comparison test (*P* value ≤ 0.05).

### Protein identification

Selected proteins which were statistically significant were cut from the gels, destained, reduced, and alkylated using dithiothreitol and iodoacetamide solutions. Next, the gel fragments were digested with trypsin solution in 50 mM bicarbonate buffer at 37 °C for 12 h (Promega, Trypsin Gold, Mass Spectrometry Grade, Technical Bulletin). The resulting peptides were eluted from the gel pieces with a water/acetonitrile/TFA solution (v:v 450:500:50) by triple extraction. Peptide mixtures were purified and concentrated using C18 Zip-TIP pipette tips according to the manufacturer’s guidelines (Merck Chemicals, Billerica, MA, USA, PR 02358, Technical Note). Next, the peptide solutions and standard solution (Peptide Calibration Standard II, Bruker, Bremen, Germany) were spotted on an Anchor Chip MALDI plate (Bruker, Bremen, Germany) and covered with 1 µL of α-cyano-4-hydroxycinnamic acid matrix (HCCA, Bruker, Bremen, Germany). Mass spectra were obtained in positive reflector mode within the 700–4000 m/z range using an Ultraflextreme MALDI TOF/TOF spectrometer (Bruker, Bremen, Germany) and flexControl 3.3 software (Bruker, Bremen, Germany). The resulting spectra were smoothed and baseline corrected. The peak list generated in flexAnalysis 3.0 software (Bruker, Bremen, Germany) for a signal-to-noise ratio of > 3 was transferred to BioTools 3.2 (Bruker, Bremen, Germany) and compared to the Swiss-Prot database (www.uniprot.org) in Mascot 2.2 software (Matrix Science, Boston, MA, USA), restricted to ‘mus musculus’, with maximum error of 0.3 Da and carbamidomethylation of cysteine as an obligatory modification. Results with a Mascot score above 55 were considered statistically significant (*P* ≤ 0.05); otherwise, the combined ion spectra of selected peptides were obtained using the LIFT mode and subjected to MALDI TOF/TOF identification.

### Statistical analysis

**Fig. 1:**
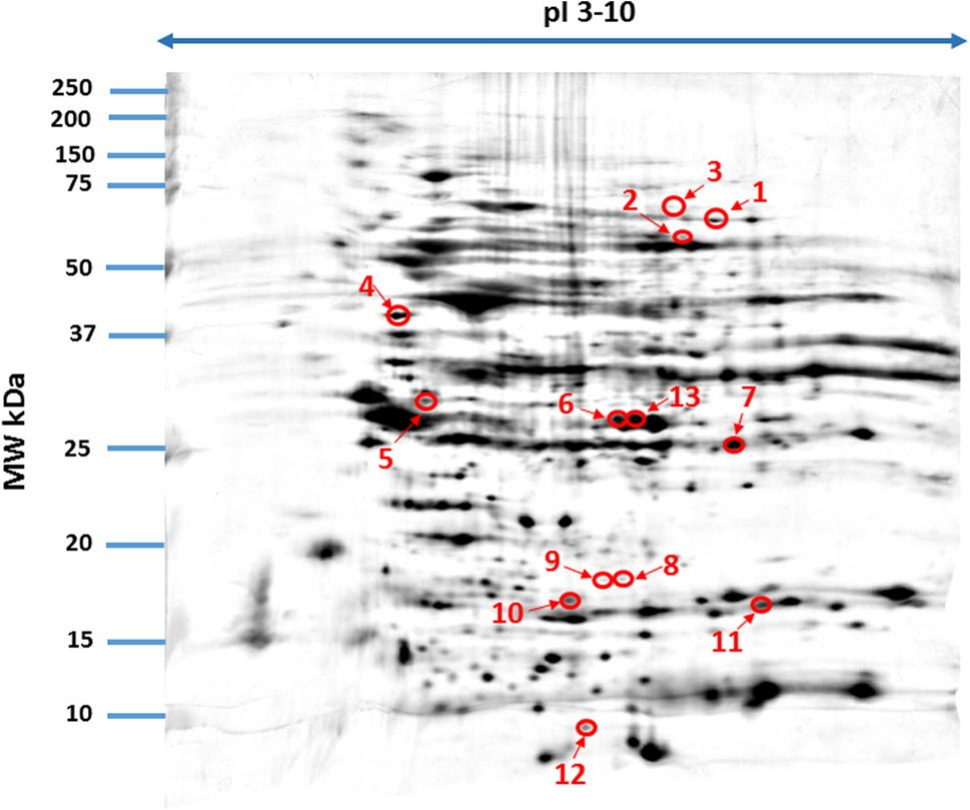
Fused image showing condensed spot patterns from the experiment. The differentially expressed proteins are marked with circles. Proteins were separated in the first dimension by isoelectric focusing over the isoelectric point (pI) range 3–10. The second dimension was performed using a 12.5% sodium dodecyl sulphate polyacrylamide gel. Gels were silver stained, digitized, and processed in Delta2D software (version 4.7 DECODON Greifswald, Germany)

**Fig. 2:**
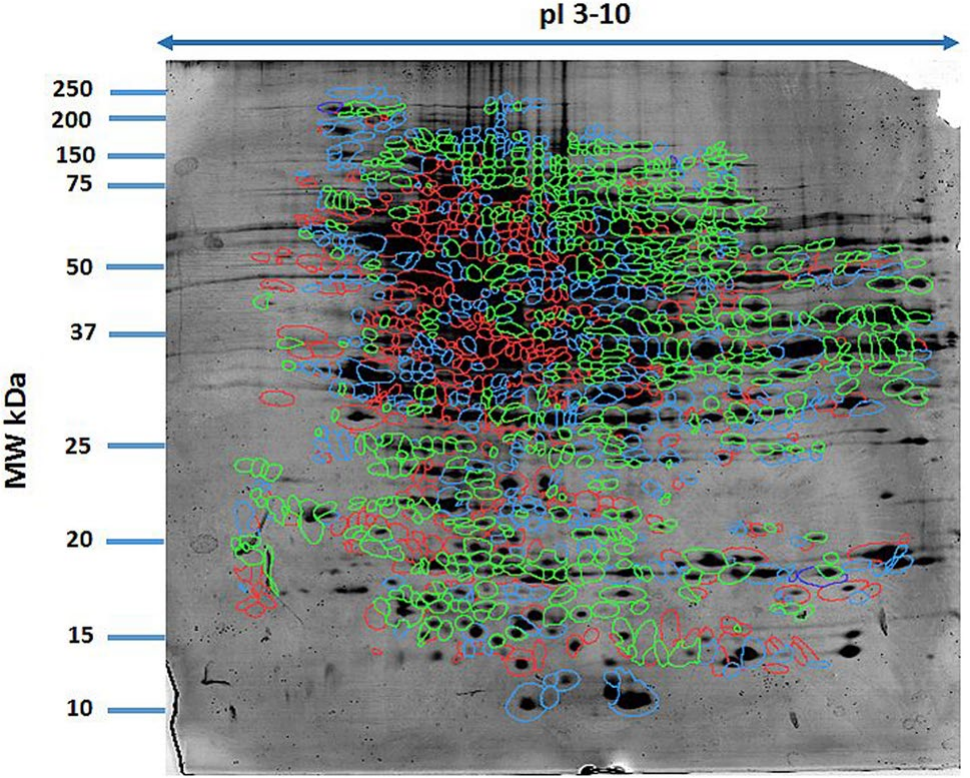
Colour coding of maximum volume of given spot according to group: green – I, red – II, blue – III. Gels were silver-stained, digitized, and processed in Delta2D software (version 4.7 DECODON Greifswald, Germany)

**Fig. 3:**
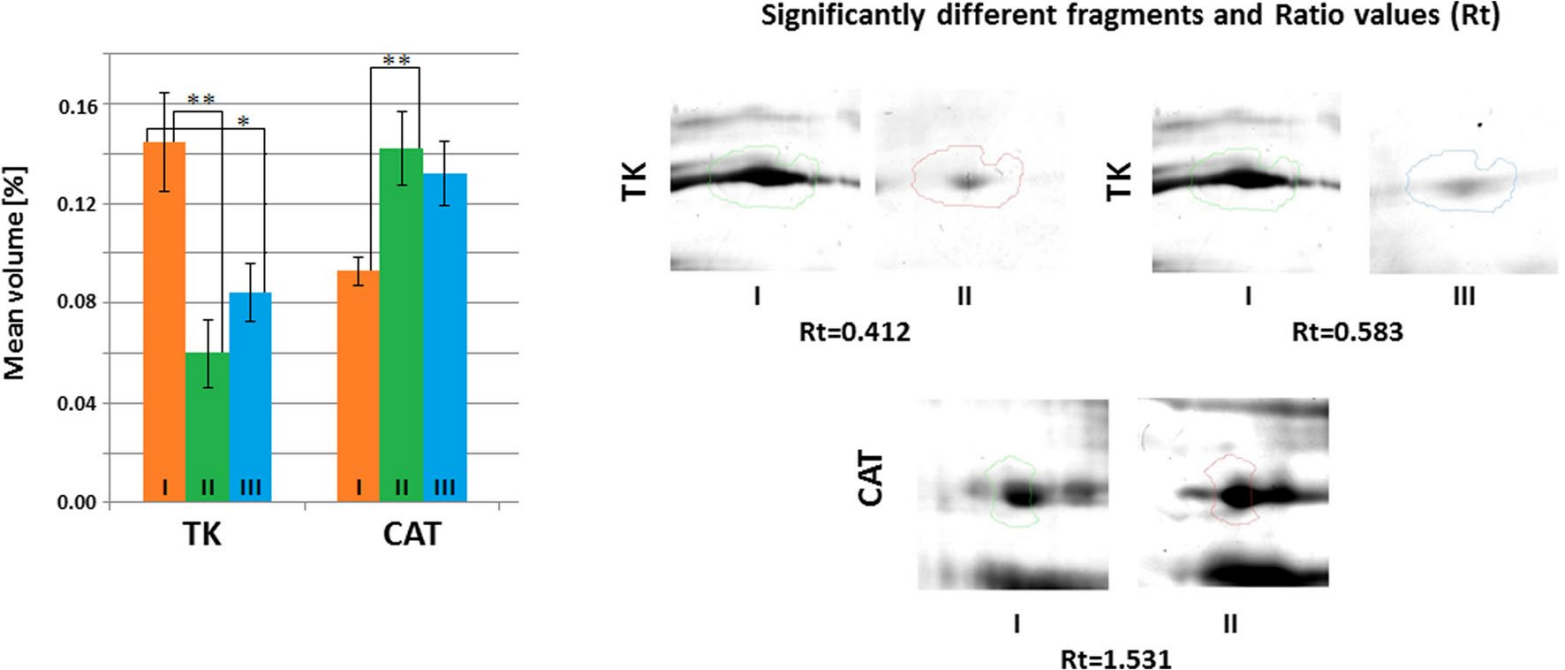
Mean volumes (%) of protein spots in the experimental and control groups. Statistically different proteins are compared, and ratio parameters (Rt) are given. TK, transketolase; CAT, catalase. Significant differences assessed using the ANOVA test and Tukey’s post hoc test are marked with asterisks: *P ≤ 0.05; **P ≤ 0.01

**Fig. 4:**
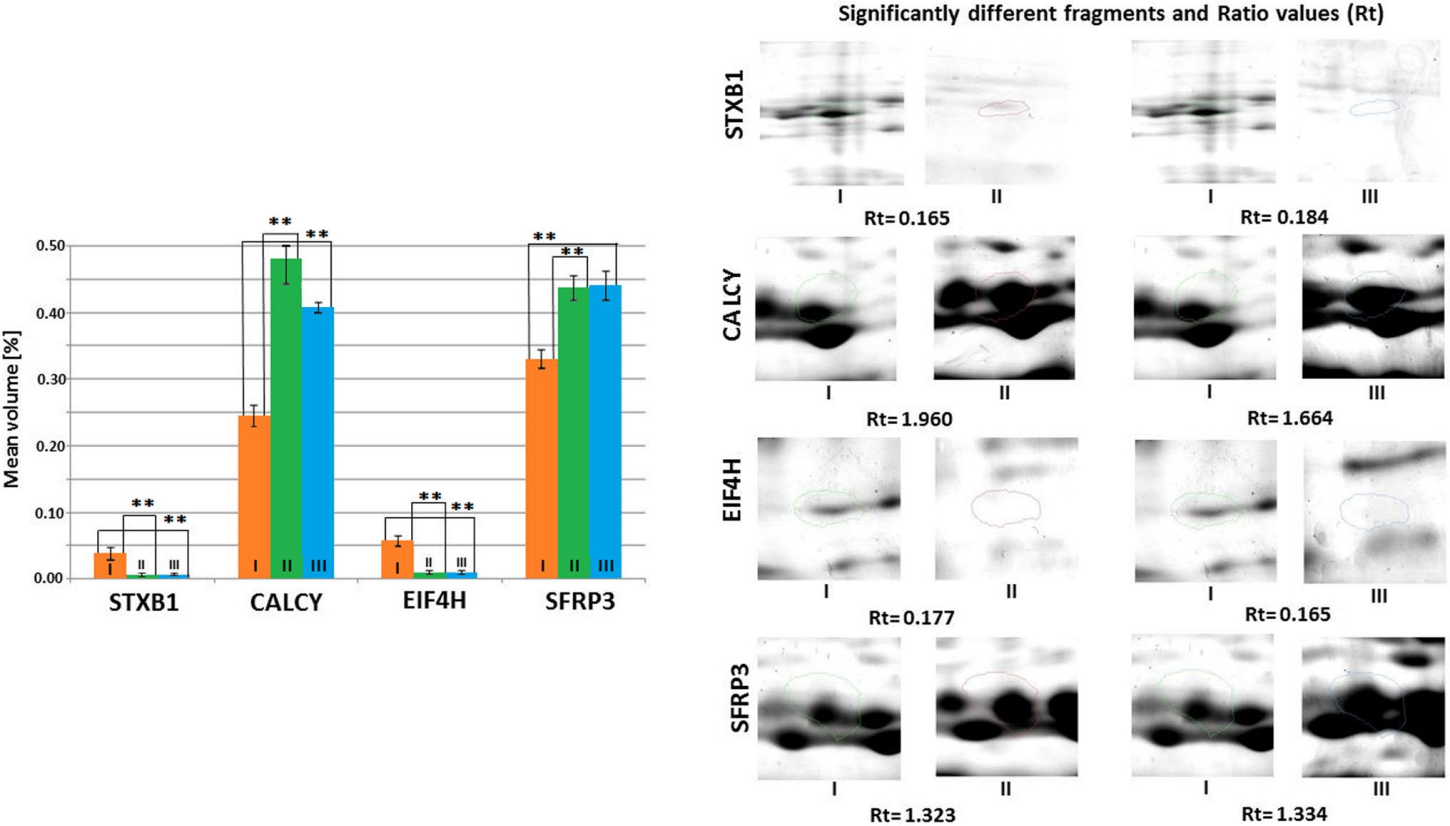
Mean volumes (%) of protein spots in the experimental and control groups. Statistically different proteins are compared, and ratio parameters (Rt) are given. STXB1, syntaxin-binding protein 1; EIF4H, eukaryotic translation initiation factor 4H; CALCY, neuron-specific vesicular protein calcyon; SFRP3, secreted frizzled-related protein 3. Significant differences assessed using the ANOVA test and Tukey’s post hoc test are marked with asterisks: *P ≤ 0.05; **P ≤ 0.01

**Fig. 5:**
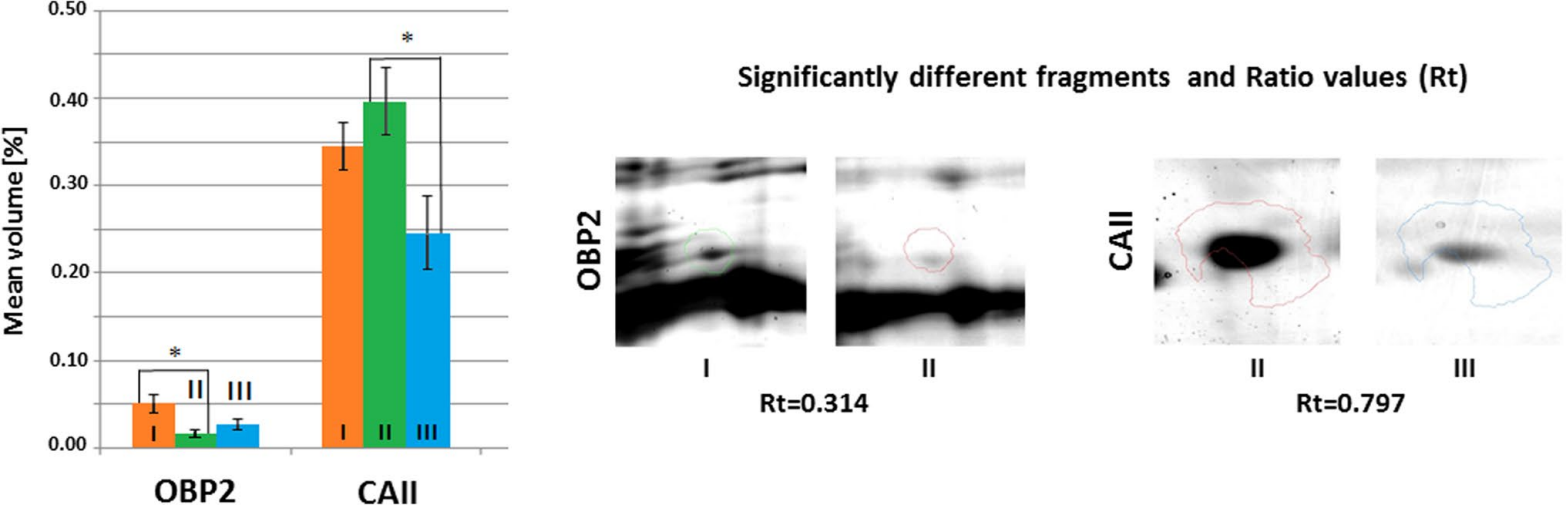
Mean volumes (%) of protein spots in the experimental and control groups. Statistically different proteins are compared, and ratio parameters (Rt) are given. OBP2, odorant-binding protein 2; CAII, carbonic anhydrase 2. Significant differences assessed using the ANOVA test and Tukey’s post hoc test are marked with asterisks: *P ≤ 0.05; **P ≤ 0.01

**Fig. 6:**
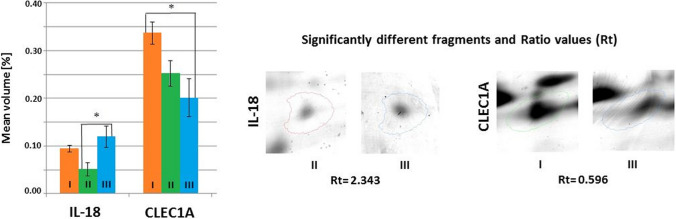
Mean volumes (%) of protein spots in the experimental and control groups. Statistically different proteins are compared, and ratio parameters (Rt) are given. IL-18, interleukin-18; CLEC1A, C-type lectin domain family 1 member A. Significant differences assessed using the ANOVA test and Tukey’s post hoc test are marked with asterisks: *P ≤ 0.05; **P ≤ 0.01

**Fig. 7:**
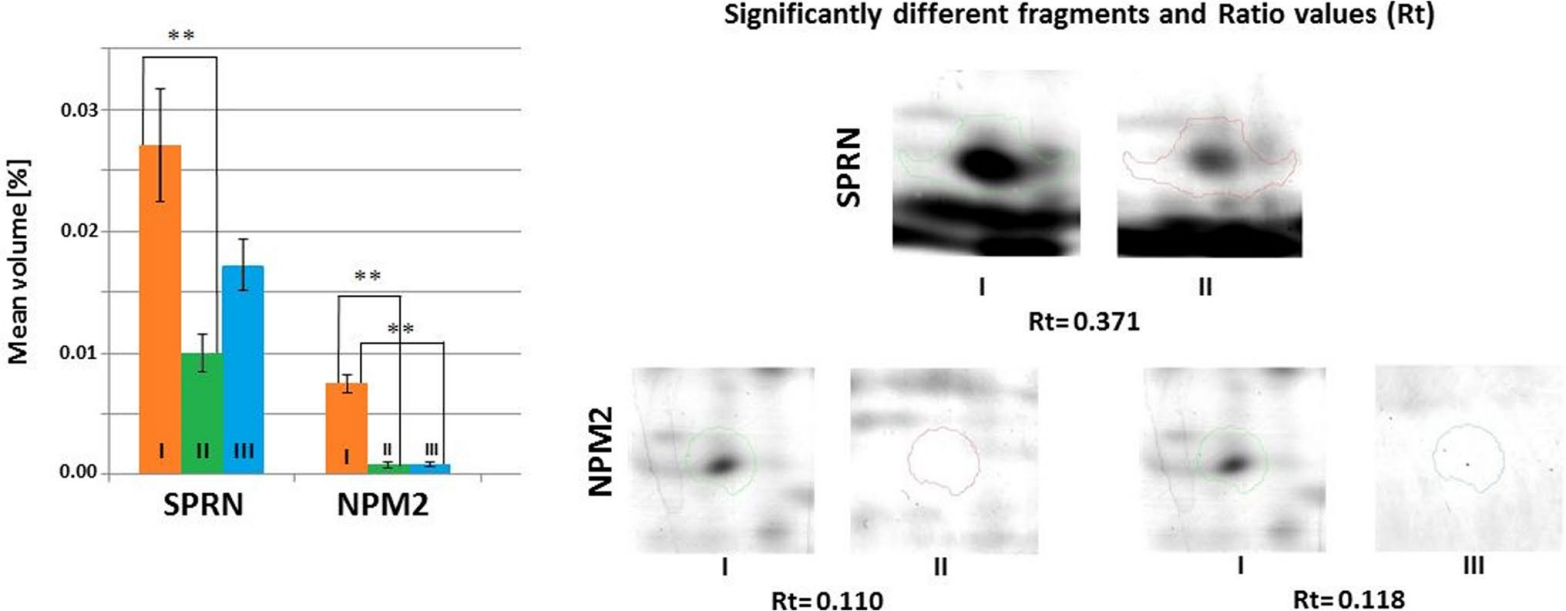
Mean volumes (%) of protein spots in the experimental and control groups. Statistically different proteins are compared, and ratio parameters (Rt) are given. SPRN, shadow of prion protein; NPM2, nucleoplasmin-2. Significant differences assessed using the ANOVA test and Tukey’s post hoc test are marked with asterisks: *P ≤ 0.05; **P ≤ 0.01

**Fig. 8:**
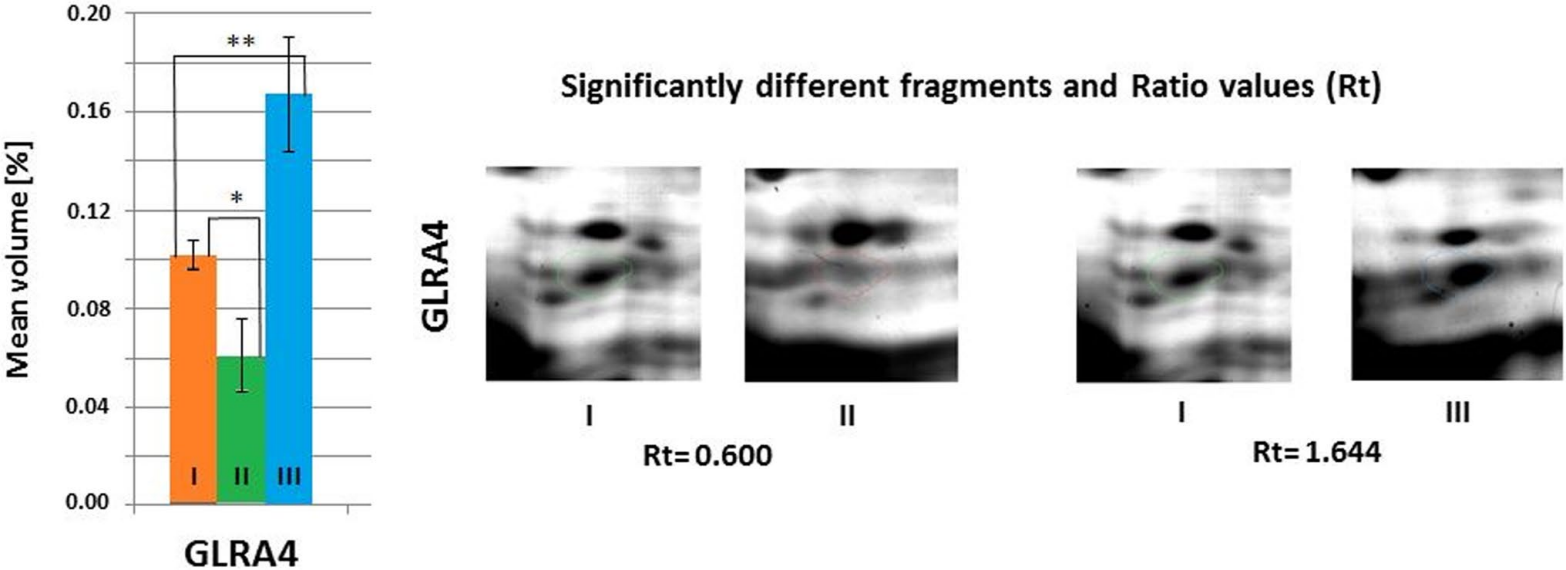
Mean volumes (%) of protein spots in the experimental and control groups. Statistically different proteins are compared, and ratio parameters (Rt) are given. GLRA4, glycine receptor α4 subunit. Significant differences assessed using the ANOVA test and Tukey’s post hoc test are marked with asterisks: *P ≤ 0.05; **P ≤ 0.01

On the basis of spot volumes, differences in protein expression between the test groups were analysed by one-way analysis of variance ANOVA with alpha critical value *P* ≤ 0.05 based on F-distribution in Delta2D 4.7.0 (DECODON, Greifswald, Germany) and a post hoc Tukey comparison test. A spot intensity ratio higher than 1.3 (upregulated) or lower than 0.67 (downregulated) was the basis for protein identification. The results were presented in Figs. 1, 2, 3, 4, 5, 6, 7, and 8 and Table 1.

**Table 1:** Differentially expressed proteins in mouse hippocampus identified by MALDI-TOF MS

ID	Protein	Accesion number (UniProtKB)	Score	Match	MW (Da)*	pI*	Modif	Seq. Cov (%)	Anova *P* value	Tukey’s HSD	Tukey’s HSD p value	Rt
1	Transketolase	P40142	75	10	68,272	7.23	C, O	18	0.0077	Cont-BB Cont-LB	0.00704 0.0544	0.412 0.583
2	Odorant-binding protein 2	Q8K1H9	123	7	20,154	5.98	C, O	19	0.0203	Cont-BB	0.0180	0.314
3	Syntaxin-binding protein 1	O08599	148	12	67,925	6.49	C, O	18	0.0042	Cont-BB Cont-LB	0.0083 0.0096	0.165 0.184
4	Catalase	P24270	79	6	60,043	7.72	C	10	0.0377	Cont-BB	0.0039	1.531
5	Glycine receptor subunit alpha-4	Q61603	103	7	52,879	8.66	C	11	0.0029	Cont-BB Cont-LB	0.053 0.0022	0.600 1.644
6	Secreted frizzled-related protein 3	P97401	114	7	37,070	8.64	C	10	0.0019	Cont-BB Cont-LB	0.0049 0.0038	1.323 1.334
7	Components: Triosephosphate isomerase Carbonic anhydrase 2	P17751 P00920	127 64	12 10	27,038 29,129	6.90 6.49	C C	67 46	0.0487	BB-LB	0.0426	0.797
8	Nucleoplasmin-2	Q80W85	83	6	23,523	5.07	C, O	15	0.0001	Cont-BB Cont-LB	0.0001 0.0001	0.110 0.118
9	Eukaryotic translation initiation factor 4H	Q9WUK2	126	8	27,381	6.67	C, O	28	0.0001	Cont-BB Cont-LB	0.00007 0.00005	0.177 0.165
10	Shadow of prion protein	Q8BWU1	81	4	14,810	10.38	C, O	29	0.0089	Cont-BB	0.0068	0.371
11	C-type lectin domain family 1 member A	Q8BWY2	99	6	31,565	7.42	C	13	0.0354	Cont-LB	0.02961	0.596
12	Interleukin-18	P70380	72	4	22,349	4.64	C, O	12	0.03736	BB-LB	0.0313	2.343
13	Neuron-specific vesicular protein calcyon	Q9DCA7	54	3	24,849	5.77	C, O	11	0.000037	Cont-BB Cont-LB	0.000032 0.001278	1.960 1.664

Abbreviations: *C* carbamidomethylation of cysteine, *O* oxidation of methionine, Match, the number of overlapping peptides in the database; Score, protein score is − 10*Log(P), where *P* is the probability that the observed match is a random event. Protein scores greater than 55 are significant (*P* < 0.05)

Listed molecular weights and pI values correspond to the MASCOT Search Result. Significant differences (*P* ≤ 0.05) were assessed by ANOVA and Tukey’s post hoc test. BB, *Bifidobacterium longum* Rosell®-175; LB, *Lactobacillus rhamnosus* JB-1

## Results

### Protein identification

Among the proteins in the samples analysed, we focused only on those with differential electrophoretic spots. MALDI-TOF mass spectrometry identified 13 statistically significant proteins (Table 2). Stains were positively identified as the enzyme transketolase (TK); odorant-binding protein 2 (OBP2), a small extracellular protein of the lipocalin superfamily; the regulator protein syntaxin-binding protein 1 (STXB1); the enzyme catalase (CAT); the transport protein glycine receptor α4 subunit (GlRA4); secreted frizzled-related protein 3 (SFRP3); the enzyme carbonic anhydrase 2 (CAII); the thermostable acidic protein nucleoplasmin-2 (NPM2); eukaryotic translation initiation factor 4H (EIF4H); shadow of prion protein (SPRN); C-type lectin domain family 1 member A (CLEC1A); interleukin-18 (IL-18); and neuron-specific vesicular protein calcyon (CALCY). Table 1 lists protein names, UniProt base accession numbers, ANOVA *P* values and Tukey’s HSD P value. See also Figs. 1 and 2 and Table 2.

**Table 2:** Summary of the key function of differentially expressed proteins in the hippocampus of mice supplemented with *Bifidobacterium longum* Rosell®-175 or *Lactobacillus rhamnosus* JB-1

Protein	Abbreviation	Function	References
Transketolase	TK	A thiamine-dependent enzyme involved in the pentose phosphate pathway which synthesizes ribose-5-phosphate and NADPH	Zhao et al. (2014)
Odorant-binding protein 2	OBP2	A soluble carrier protein involved in olfactory transduction with the strongest affinity for long-chain aldehydes and fatty acids	Tcatchoff et al. (2006)
Syntaxin-binding protein 1	STXB1	Protein involved in neurotransmission, it takes part in docking, priming, and fusion of the synaptic vesicles through interactions with SNAREs	Chen et al. (2020)
Catalase	CAT	A key antioxidant enzyme that catalyzes hydrogen peroxide to water and molecular oxygen and protects the tissues from highly reactive hydroxyl radicals	Weydert and Cullen (2010)
Glycine receptor subunit alpha-4	GLRA4	One of the subunits of GlyR which plays an essential role in inhibitory neurotransmission; the exact function of α4 subunit is not known as the human ortholog is considered a pseudogene. In mice, GlyR α4-mediated glycinergic neurotransmission may modulate social, startle, and anxiety-like behaviour	Darwish et al. (2023)
Secreted frizzled-related protein 3	SFRP3	A modulator of the Wnt signalling pathway that among others regulates diverse developmental processes in the embryonic brain and adult hippocampal neurogenesis	Jang et al. (2013a, b)
Carbonic anhydrase 2	CAII	An enzyme that reversibly converts carbon dioxide and water to bicarbonate and H^+^, it is involved in the regulation of pH homeostasis in the brain	Boone et al. (2013), Lemon et al. (2021)
Nucleoplasmin-2	NPM2	A protein involved in chromatin condensation, it plays important role during initial stages of embryonic development but the function of this protein in the brain remains unknown	Burns et al. (2003), Lingenfelter et al. (2011)
Eukaryotic translation initiation factor 4H	EIF4H	A factor involved in protein synthesis at the level of initiation phase, necessary for the proper development and brain morphology	Capossela et al. (2012)
Shadow of prion protein	SPRN	A PrP-like protein, its role is not fully known. It may be involved in the control of cellular proliferation and differentiation of various tissues	Passet et al. (2020)
C-type lectin domain family 1 member A	CLEC1A	A member of CLR receptors family involved in the defence against pathogens, it can be involved in the development of CNS inflammation by up-regulating transport of Th17 cells through the blood–brain barrier	Makusheva et al. (2022)
Interleukin-18	IL-18	A pro-inflammatory cytokine, an important mediator of inflammation and host immune response. It also modulates neuronal functions, mediates communication between the CNS and PNS, and regulates the activity of the hypothalamic–pituitary axis	Alboni et al. (2010), Kuwahara-Otani et al. (2017)
Neuron-specific vesicular protein calcyon	CALCY	A transmembrane protein that interacts with clathrin, stimulates clathrin assembly, and clathrin mediated endocytosis	Muthusamy et al. (2012)

*CLR* C-type lectin receptor, *CNS* central nervous system, *GlyR* glycine receptor, *NADPH* reduced form of nicotinamide adenine dinucleotide phosphate, *PNS* peripheral nervous system, *PrP* prion protein, *SNAREs*, neuronal soluble *N*-ethylmaleimide-sensitive factor-attachment protein receptors

### Protein profile of the hippocampus

The results indicate statistically significantly higher (*P* ≤ 0.05) concentrations of TK in the hippocampus of mice in the control group compared to both experimental groups. The mean volume of CAT was statistically significantly higher (*P* ≤ 0.05) in the hippocampus of mice receiving a *Lactobacillus rhamnosus* JB-1 suspension compared to the control group. No statistically significant differences were observed in the mean volume of catalase in the group of mice receiving the *Bifidobacterium longum* Rosell®-175 suspension compared to the control group (Fig. 3).

Statistically significantly lower (*P* ≤ 0.05) mean volumes of STXB1 and EIF4H were observed in the hippocampus of mice in both experimental groups compared to the control group. The mean volumes of CALCY and SFRP3 in the hippocampus of mice from both experimental groups were statistically significantly higher (*P* ≤ 0.05) than in the control group. However, there were no statistically significant differences in the mean volumes of these proteins between the two experimental groups (Fig. 4).

In the case of CALCY, it was not possible to obtain statistically significant identification results with the MALDI TOF method. A score of 54 was achieved (a score above 55 is necessary for statistically significant identification). Nevertheless, three peptide matches were confirmed during tandem mass spectrometry in triplicate. Statistically significant identification may have been prevented by the considerable post-translational modifications resulting from psychobiotics administration.

The mean volume of OBP2 in the group of mice receiving the *Lactobacillus rhamnosus* JB-1 suspension was statistically significantly lower (*P* ≤ 0.05) than in the control group. However, the mean volume of CAII was statistically significantly lower (*P* ≤ 0.05) in the tissues of mice receiving the *Bifidobacterium longum* Rosell®-175 suspension compared to the group of mice receiving the *Lactobacillus rhamnosus* JB-1 suspension (Fig. 5).

No statistically significant differences in the mean volume of IL-18 were observed between the control and experimental groups. However, the mean volume of this protein was statistically significantly higher (*P* ≤ 0.05) in the group of mice receiving the *Bifidobacterium longum* Rosell®-175 suspension compared to the group supplemented with the *Lactobacillus rhamnosus* JB-1 suspension. The mean volume of CLEC1A was statistically significantly higher (*P* ≤ 0.05) in the control group compared to the group supplemented with *Bifidobacterium longum*. However, no statistically significant differences in the mean volume of this protein were observed between the experimental groups or between the *Lactobacillus rhamnosus* JB-1-supplemented group and the control group (Fig. 6).

Compared to the control group, statistically significantly lower (*P* ≤ 0.05) mean volume of shadow of prion protein (SPRN) was observed in the hippocampus of mice from the *Lactobacillus rhamnosus* JB-1 supplemented group. Similarly, significantly lower mean volume of NPM2 was observed in both experimental groups compared to the control group. No statistically significant differences in mean volumes of NPM2 were observed between the experimental groups (Fig. 7).

Statistically significant differences (*P* ≤ 0.05) in the mean volume of GLRA4 were observed in the hippocampus of mice in both experimental groups compared to the mean volume of this protein in the tissues of mice from the control group. The obtained results indicate that the average volume of GLRA4 in the hippocampus of mice from the group supplemented with *Lactobacillus rhamnosus* JB-1 was statistically significantly lower (*P* ≤ 0.05) compared to the average volume of these proteins in mice from the control group. However, in the group of mice supplemented with *Bifidobacterium longum* Rosell®-175, a statistically significantly higher (*P* ≤ 0.05) mean volume of GLRA4 was observed compared to the control group (Fig. 8).

## Discussion

**Fig. 9:**
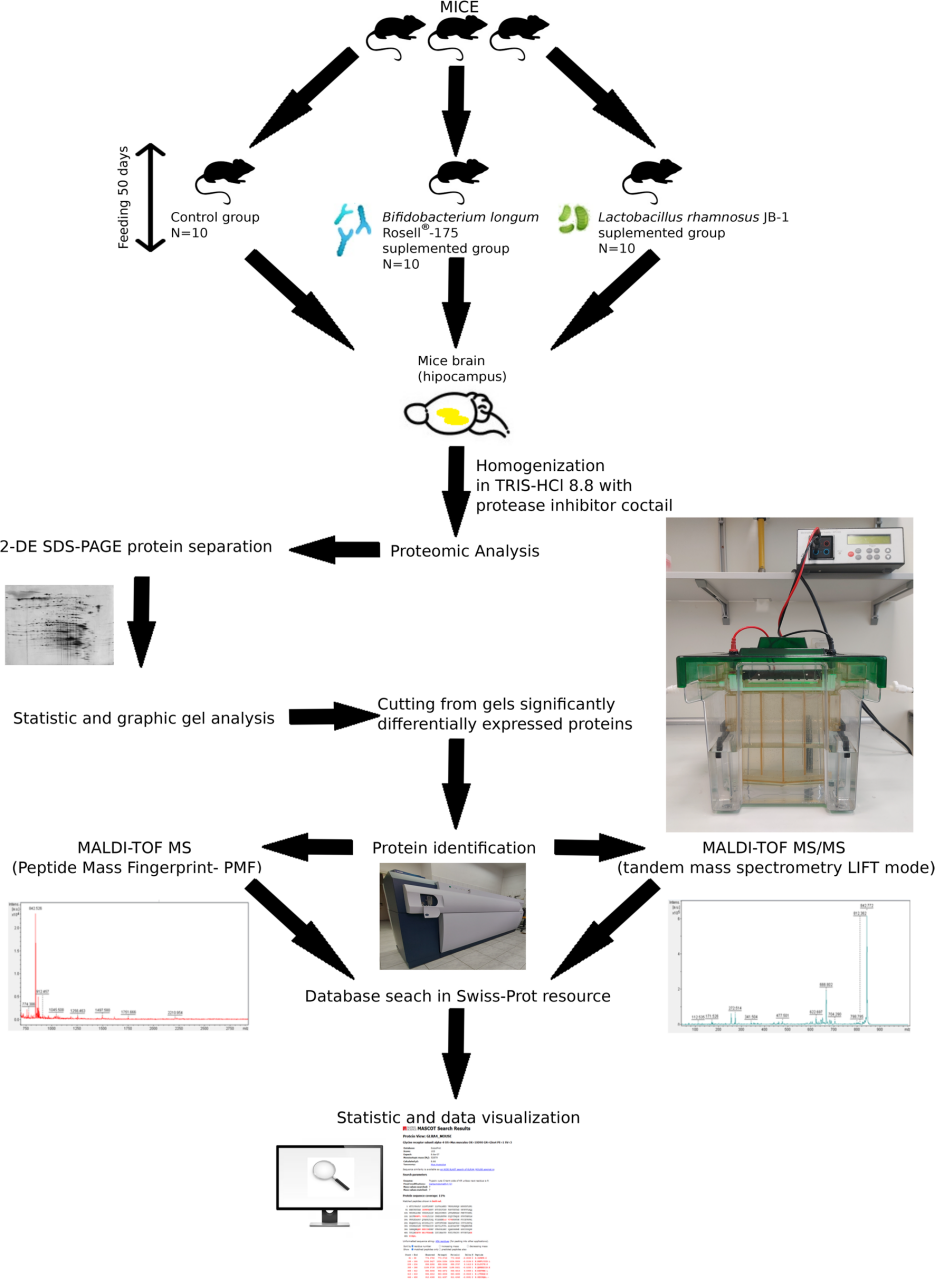
Diagram of the experiment and the tests performed

The study provided the first evidence of differentiation of the proteomic profile of the hippocampus of mice in response to diet supplementation with *Lactobacillus rhamnosus* JB-1 and *Bifidobacterium longum* Rosell®-175. The proteins which differentiate mice from the experimental groups and the control perform numerous functions in the body, taking part in important cellular processes (Fig. 9, Tables 1 and 2).

Changes were shown in the expression of SFRP3, a secreted frizzled-related protein. SFRPs are a family of soluble proteins with the ability to inhibit signalling pathways by binding to Wnt ligands and/or Fz receptors (serpentine receptors, called frizzleds) (Bovolenta et al. 2008). It should be noted that in various experimental models SFRP3 acts as an antagonist or agonist of the Wnt signalling pathway, causing an increase in the concentration of this protein in states of tumour progression and metastasis (Pećina-Šlaus et al. 2016). Wnt signalling regulates brain development processes during the embryonic period and controls proliferation and differentiation of progenitor cells in the nervous system in the postnatal period (Patapoutian and Reichardt 2000; Shu et al. 2018). Neurogenesis in the hippocampus proceeds in parallel with peripheral neurogenesis and is regulated by physiological and pathological stimuli, which influence neuron activity (Shohayeb et al. 2018; Fares et al. 2019). Stimuli accelerating neuronal maturation and integration include physical activity, learning processes, and the occurrence of pathological seizures, as well as the psychobiotics *L. rhamnosus* JB-1 and *B. longum* Rosell®-175 used in the study, which stimulate synthesis of neuroactive proteins through the microbiota–gut–brain axis. The increase in the expression of these proteins in both experimental groups may suggest that they could be involved in the activation and maturation of nerve cells. Previous research indicates that bacteria such as *L. rhamnosus* strain GG modulate Wnt/β-catenin signalling pathways in various cell lines, including cancer cells, increasing expression of SFRP (Aherian-Esfahani et al. 2016). The increase in the expression of SFRP3 in the present study following administration of *B. longum* and *L. rhamnosus* may also be related to the regulation of the expression of genes of Wnt-associated signalling pathways. Wnt signalling plays an important role in regulating early brain development, cell migration, dendrite morphogenesis, and synapse formation, as well as in controlling cognitive functions, thus preventing neurodevelopmental, neurological, and neurodegenerative disorders (Hussaini et al. 2014; Seib et al. 2013). The latest data suggest that SFRP3 is an endogenous antagonist of Wnt, and a decrease in its secretion stimulates neurogenesis in the hippocampus (Jang et al. 2013a, b) and promotes antidepressant activity in mice and humans (Jang et al. 2013a). Nevertheless, the concentration of SFRP3 mRNA in the mouse hippocampus remains unchanged for its entire life (Jang et al. 2013a, b; Cho et al. 2019) and exerts a neuroprotective effect by promoting myelination. The results of our experiment indicate the need for continued research to assess the effect of SFRP3 on processes of cellular signalling, myelination, and neurogenesis, which is crucial to explaining the mechanisms and processes associated with neurodevelopmental disorders and to the development of therapeutic strategies to prevent a decline in cognitive functions.

In our experiment, the psychobiotics *Lactobacillus rhamnosus* JB-1 and *Bifidobacterium longum* Rosell®-175 also significantly affected CALCY. Previous research indicates that CALCY plays an important role in brain function and in the development of psychological disorders by influencing neuron development and synaptic plasticity (Li et al. 2011; Chander et al. 2019). Specifically, it is involved in endocytosis within the synapses, mediated by clathrin, which is essential to synaptic transmission and optimization of the range of released neurotransmitters, as well as in dopamine-related signalling and dopamine activity (Xiao et al. 2006). Neurotransmission processes associated with dopamine affect various brain functions, such as motor control and cognitive processes (Seamans and Yang 2004). Intestinal bacteria, including the psychobiotics used in the experiment, have been shown to produce numerous neurotransmitters in metabolic processes, including dopamine, noradrenaline, serotonin, GABA, and acetylcholine (Lyte 2011). Liu et al. (2016) demonstrated that administration of *Lactobacillus plantarum* PS128 to mice increases dopamine and serotonin concentrations in the prefrontal cortex and striatum. Therefore, it is possible that the psychobiotics used in the experiment, by producing neurotransmitters and stimulating expression of CALCY, influence synaptic transmission and coordinate signal processing and mechanisms of intercellular communication in the central and peripheral nervous system. A precise understanding of the mechanisms underlying these phenomena requires in-depth immunohistochemical and metabolic analysis of the functioning of the mouse CNS.

The study showed that administration of *Lactobacillus rhamnosus* JB-1 and *Bifidobacterium longum* Rosell®-175 to mice as diet supplements also alters expression of isoform II of carbonic anhydrase (CAII). Carbonic anhydrases (CA) are a large group of zinc metalloenzymes present in mammals in 14 different isoforms and catalysing reversible hydration of carbon dioxide to bicarbonate (Boone et al. 2013). They perform various functions in the body, including regulation of the water and electrolyte balance and pH homeostasis and a role in numerous metabolic pathways, such as gluconeogenesis, lipogenesis, and ureagenesis. In addition, they take part in bone resorption and calcification and in the formation of cerebrospinal fluid. One of the cytosolic isoenzymes of CA is the isoform CA II, expressed in the CNS (Lemon et al. 2021). CA II is present in the myelin and glial cells, microglia, choroidal epithelium, astrocytes and neurons, and in mice primarily in the oligodendrocytes and myelin sheaths (Lakkis et al. 1997). CA activity is involved in the regulation of extracellular pH in the brain, which affects neuronal activity. The increase in the expression of this protein in mice receiving *B. longum* Rosell®-175 may indicate effective modulation of the pH of the extracellular fluid, which contributes to generation of optimal synaptic currents and strengthens and maintains the metabolic activity of neurons. It is worth noting that administration of compounds such as amino acids to mice increases CA activity in the brain, thereby improving memory. Our results suggest that the increased expression of CAII in the hippocampus following administration of *B. longum* Rosell®-175 to mice stimulates activation of the brain through memory formation, processing and enhancement. In addition, Giacobini (1987) suggest that high CA concentrations are favourable in the early stages of neuron growth and maturation, and thus the high expression of this protein in the group of mice receiving *B. longum* Rosell®-175 may be linked to neuronal development and the acquisition of functions by CNS cells. Precise knowledge of the mechanisms underlying these phenomena requires in-depth research on the effects of psychobiotics on the functioning of the CNS of mice, including the hippocampus.

Analysis of the results of the study indicated decreased expression of OBP2 following the use of *Lactobacillus rhamnosus* JB-1 and *Bifidobacterium longum* Rosell®-175 in the diet of mice. OBP in vertebrates is expressed in the nasal epithelium, where it mediates olfactory transduction, binding and transporting hydrophobic and volatile aromatic molecules and pheromones through watery mucus (Pelosi et al. 2005; Forêt and Maleszka 2006). Binding of aromatic substances involves OBP2, a soluble carrier protein which shows the strongest affinity for long-chain aldehydes and fatty acids (Tcatchoff et al. 2006). The mRNA encoding this protein is more strongly expressed in the tissues of the brain, heart, kidney, liver, genitals, and lungs (Yanai et al. 2005). OBP has also been shown to be expressed in the brain tissue of developing insects (Forêt and Maleszka 2006), transporting ligands of various molecules for neuron development and signal transmission, thereby influencing insect behaviour, including adaptation to the environment (Forêt and Maleszka 2006; Guo et al. 2018). OBP has also been shown to exert broad-spectrum antimicrobial effects (Bianchi et al. 2019). The lower expression of this protein in the hippocampus of mice from the experimental groups does not rule out an analogous effect in the case of the microbes used in our study. This phenomenon may be explained by different metabolic pathways in which these microbes are involved, whereby metabolic products other than fatty acids and aldehydes which strongly stimulate OBP are supplied to the CNS.

All biological processes, including metabolic transformations, entail the generation of reactive oxygen species (ROS) and free radicals, which are responsible for the development of oxidative stress (Lushchak 2014). ROS are essential for the functioning of the body, playing the role of signal transmitters, regulating repair processes in cells and gene expression, and taking part in metabolism and redox reactions (Dröge 2002). Excessive ROS cause oxidative stress, leading to damage to cell components and disturbing cellular integrity (Schieber and Chandel 2014). Oxidative stress and ROS play an important role in neurological disorders and age-related cognitive performance, as shown in studies in humans (Dröge 2002; Mariani et al. 2005; Singh et al. 2019). These compounds also modulate synaptic transmission processes (Knapp and Klann 2002; Serrano and Klann 2004) and take part in signalling pathways (D'Autréaux and Toledano 2007), and by acting on the amygdala and hippocampus, they influence behavioural and cognitive functions. Protection against these effects is ensured by the antioxidant system in various tissues and systems, an important component of which is the enzyme CAT. CAT neutralizes O2- anion radicals, hydroxyl radicals and radicals of unsaturated fatty acids and breaks down hydrogen peroxide formed during cellular respiration to molecular oxygen and water, ensuring a state of dynamic balance between the formation and elimination of ROS (Weydert and Cullen 2010). A high CAT concentration has been noted in the liver, kidneys, and erythrocytes, but its presence has been confirmed in the brain as well (Scaglione et al. 2016). Studies on an animal model have shown that the interaction between a probiotic administered with feed and the intestinal microbiome modulates the body’s immune response to various harmful factors, including inflammation and oxidative stress (Zhang et al. 2019). Our study showed a high concentration of this protein in the hippocampus of mice receiving a diet supplemented with the psychobiotics *Lactobacillus rhamnosus* JB-1 and *Bifidobacterium longum* Rosell®-175, which confirms that they can stimulate the gut–brain axis and provides evidence of stimulation of the antioxidant system. The efficient antioxidant system, including catalase activity, guarantees neuromodulatory function and maintenance of homeostasis and presumably affects signalling pathways and cognitive functions. This hypothesis is supported by Clausen et al. (2010, 2012) and Olsen et al. (2013), who showed that the use of antioxidants such as CAT mimetics in mice corrects or mitigates cognitive deficits and fear-conditioning deficits caused by oxidative stress and stimulates synaptic plasticity by taking part in neurotransmission. Wang et al. (2009) and Cui et al. (2012) showed that decreased CAT activity is associated with impairment of hippocampus-dependent spatial memory. The results of our study confirm the stimulatory role of psychobiotics in processes limiting stress responses by stimulating production of antioxidant enzymes. It is worth emphasizing that *Lactobacillus* strains with strong antioxidant properties occur in nature (Kono and Fridovich 1983), and these can be used as active components of probiotic supplements. One of these microbes is *L. plantarum*, which produces manganese pseudocatalase, an enzyme stimulating conversion of H_2_O_2_ to water and oxygen. The biological activity of this enzyme has been shown to be similar to that of haem CAT, which is present in cells (Kono and Fridovich 1983). While these properties have not been assessed for the strains *Lactobacillus rhamnosus* JB-1 and *Bifidobacterium longum* Rosell®-175 used in our study, this type of effect cannot be ruled out.

Analysis of the results of the study indicates that administration of *Lactobacillus rhamnosus* JB-1 and *Bifidobacterium longum* Rosell®-175 to mice leads to a reduction in the expression of proteins STXB1, EIF4H, CLEC1A, TK, SPRN, and NPM2.

Clec1A is one of the CLR receptors taking part in the host’s defence against pathogens and is also involved in regulating immune system function and the development of autoimmune and neoplastic processes (Makusheva et al. 2022). This protein also plays an important role in the development of CNS inflammation by upregulating transport of Th17 cells through the blood–brain barrier (Arima et al. 2012; Makusheva et al. 2022). The low expression of this protein in the experimental groups receiving psychobiotics may indicate that there were no ongoing inflammatory processes in the CNS, autoimmune processes, or tissue degradation. In this context, it could be interesting to note the results obtained for the expression of interleukin 18, which was higher in the experimental group receiving *Lactobacillus rhamnosus* JB-1 than in the control group, but lower in the group receiving *Bifidobacterium longum* Rosell®-175 than in the controls. IL-18 mediates mechanisms of the innate and acquired immune response and regulates the cellular and humoral immune response (Alboni et al. 2010). In the publish study’s demonstrated that, IL-18 was expressed in all structures of the brain, with the highest concentrations noted in the hypothalamus, hippocampus, and amygdala. Studies of the hippocampus in mice and rats have shown that IL-18 modulates neuronal functions, takes part in synaptic transmission, mediates communication between the central and peripheral nervous system, and regulates the activity of the hypothalamic–pituitary axis (Alboni et al. 2010; Kuwahara-Otani et al. 2017). The increased expression of IL-18 in conjunction with the results obtained for previously described proteins, e.g. CALCY, may suggest that administration of the psychobiotic *Lactobacillus rhamnosus* JB-1 to mice leads to stimulation of synaptic transmission, which influences neuromodulatory processes, memory, and cognitive functions. An increase in IL-18 expression may also be linked to inflammatory processes and brain damage (Alboni et al. 2010). It is worth emphasizing, however, that the pleiotropic role of IL-18 in CNS development is not fully understood. It is known to inhibit neuron differentiation, inducing the death of nerve cells, and to take part in the formation of neural stem cells in response to trauma (Johansson et al. 1999; Lingenfelter et al. 2011). *Lactobacillus* strains are also known to have the ability to activate MDC in order to induce an immune response in T cells and to induce the formation and secretion of Th1 cytokines, including IL-18, IFN I and II, and IL-12 (Mohamadzadeh et al. 2005). That study supports the results of our experiment, in which expression of IL-18 was increased in the group of mice receiving *Lactobacillus*.

We obtained similar results for the expression of the protein GLRA4, a glycine receptor containing subunit α4. Expression of glycine receptors (GlyR) has been shown in the developing brain and in the fully developed spinal cord, hindbrain, cerebellum, and retina. They play an important role in mediation of inhibitory neurotransmission in the brain and spinal cord, and also take part in stimulatory neurotransmission in embryonic neurons (Matzenbach et al. 1994; Harvey et al. 2000; Baer et al. 2009; Lynch 2009). Among the five GlyR subtypes, the function of GLRA4 in people and animals remains unclear, because the human GLRA4 gene is considered a pseudogene (Simon et al. 2004), present in the genome in the form of a neutral sequence with no biological function (Simon et al. 2004; Bar-Shira et al. 2015). Nevertheless, many studies indicate that pseudogenes can perform biological functions and play a role in health and disease (Tam et al. 2008; Pink et al. 2011). Given that most neurons of the CNS are inhibited by glycine (Nicoll et al. 1990), an increase in GLRA4 expression may be linked to inhibition of neurotransmission in the brain of mice. Expression of GLRA4 in the study may not fully reflect its physiological role in the hippocampus of mice following administration of the diet supplement. Increased expression of GLRA4 may be linked to brain development dependent on amino acids released from glial cells, e.g. taurine or β-alanine (Flint et al. 1998; Mori et al. 2002). GLRA4 is found in large quantities in the synapses, where it takes part in regulation of synaptic stimulation and inhibition (Legendre 2001). The synapses in the spinal cord (Jonas et al. 1998), brainstem (Russier et al. 2002), and cerebellum (Dumoulin et al. 2001) include mixed GABA/glycine synapses which can mediate neurotransmission, while activation of GlyR may inhibit GABA_A_Rs via a phosphorylation-dependent mechanism (Li et al. 2003). Results reported by Bravo et al. (2011) suggest that *Lactobacillus rhamnosus* JB-1 exerts a direct effect on the GABAergic system and neurotransmission processes in mice, reducing behaviours associated with depression and anxiety. Similarly, Yunes et al. (2020) found that *Lactobacillus plantarum* 90sk and *Bifidobacterium adolescentis* 150 produce large amounts of GABA, which modulates GABAergic signalling through GABA receptors located on intestinal neurons (Yunes et al. 2016). This demonstrated the antidepressant effect of these receptors in mice, expressed as a reduction in behaviours reminiscent of depression. The increase in GLRA4 expression in our study in the group of mice receiving *Lactobacillus* may be associated with neural transmission, which ensures the normal function of complex cerebral processes such as neuron excitability, synaptic plasticity, and cognitive functions, e.g. learning and memory. It should be noted that lactic acid bacteria (LAB) of the genus *Lactobacillus* produce large amounts of GABA during fermentation, and GABA receptors are present in the intestinal microbiota (Yunes et al. 2016). These data in combination with the results of the present study indicate an interdependence between glycine and GABA receptors, which translates to modulation of the ‘stimulation–inhibition’ balance and demonstrates the value of using psychobiotics to treat abnormal behaviour associated with anxiety and depression (Sarkar et al. 2016).

The present study also showed reduced expression of the synaptic protein STXB1, which is essential in neurotransmission processes (Chen et al. 2020). Interacting with and binding syntaxin, this protein takes part in fusion of synaptic vesicles with presynaptic membranes, releasing neurotransmitters (Shen et al. 2015). The lack of the gene encoding Stxbp1 or its low expression even results in complete loss of the release of neurotransmitters and synaptic conduction, and in consequence in brain dysfunctions (Zoghbi and Bear 2012), and various pathological states (Saitsu et al. 2008). Miyamoto et al. (2017) showed that transgenic mice with overexpression of Stxbp1 displayed increased aggression, while mice with a Stxbp1 + / − deficit exhibited impaired neurotransmission-dependent cognitive processes. These studies show that synaptic transmission deficits induced by a deficiency or lack of Stxbp1 can cause disturbances in nervous system development and induce disease and behavioural disorders. In our study, the STXB1 concentration in mice following administration of psychobiotics was statistically significantly lower than in the control group.

Low expression of EIF4H was also shown in the groups of mice receiving psychobiotics. A deficiency of this protein in mice induces growth disorders, changes in the brain in the form of neuronal morphology disorders, and behavioural disorders associated with learning and memory, indicating damage to the hippocampus and amygdala (Capossela et al. 2012; Kats and Klann 2019). Due to the lack of literature data on the function of EIF4H in health and disease, it is not possible to state whether a lack or decreased amount of EIF4H causes abnormalities in the CNS, expressed as developmental and functional disorders. EIF4H is present in all structures of the brain, including in the synapses, and is involved in protein synthesis and mRNA translation. Reduced expression of this protein affects synaptic protein synthesis, which may underlie the onset of neuronal signalling disorders and lead to changes in animal behaviour (Capossela et al. 2012). Further research on the role of EIF4H in neuronal development and CNS functioning is needed.

Proteins for which we showed no differences in expression between the experimental groups and the control also include NPM2 and SPRN. The physiological function of SPRN in the CNS is not fully known. SPRN is a protein with properties similar to those of PrPc, so it is presumed that it may also take part in the development of neurodegenerative diseases in humans, such as Alzheimer’s disease (Passet et al. 2020). In mice, this protein is present in two regions of the brain—in the Purkinje cells in the cerebellum and in the pyramidal cells of the hippocampus, and an increase in its expression is linked to numerous functions performed during embryonic development and to tissue growth and development (Lloyd et al. 2009). NPM2 is involved in chromatin condensation, and a deficiency of this protein causes developmental defects and embryo mortality (Burns et al. 2003). However, the exact function of this protein in the CNS of mice has not yet been described. Low expression of both proteins demonstrates that they are mainly responsible for cell development during the embryonic period, and their concentrations are higher in reproductive cells and organs (Lingenfelter et al. 2011). An increase in the concentrations of these proteins may be associated with the development of diseases impairing neuron function and morphology, which may be linked to pathological cell proliferation.

Vitamins take part in many physiological and biochemical processes. One such vitamin is thiamine (vitamin B_1_), a deficiency of which causes metabolic disorders associated with a lack of production of enzymes such as pyruvate dehydrogenase and α-ketoglutarate dehydrogenase complex (Whitfield et al. 2018). Adequate thiamine intake is also essential for the CNS, as it performs neuromodulatory functions in the acetylcholine neurotransmitter system and takes part in the structure and functions of cell membranes, including of neurons and neuroglia (Mkrtchyan et al. 2015). Converted to thiamine pyrophosphate, thiamine also acts as a coenzyme for TK. TK is the key enzyme in the pentose phosphate pathway (PPP), which synthesizes ribose-5-phosphate and the reduced form of nicotinamide adenine dinucleotide phosphate (NADPH) (Zhao et al. 2014). Thiamine deficiency has been shown to reduce transketolase activity, which impairs the energy metabolism of the cell, reduces the viability of brain cells, and contributes to the onset of neurodegenerative diseases (Liu et al. 2017; Håglin et al. 2020) and neurological disorders associated with reduced behavioural and cognitive functions. The experiment showed a very low concentration of this enzyme in the hippocampus of mice receiving *Lactobacillus rhamnosus* JB-1 and *Bifidobacterium longum* Rosell®-175 compared to controls. Research on the effect of thiamine-producing lactic acid bacteria (LAB) on the intestinal microbiota, the microbiota–gut–brain axis, and neuron metabolism in the CNS has demonstrated that *L. rhamnosus* is able to produce thiamine intracellularly (Teran et al. 2021). This suggests that these bacteria could be used as an alternative to pharmaceuticals to support the treatment and prevention of neurodegenerative diseases. However, none of the bacterial strains studied thus far has led to the production of an adequate concentration of thiamine (Masuda et al. 2012), as confirmed by our own results. The low content of TK in the hippocampal cells may indicate increased demand for aerobic metabolism and synthesis of neurotransmitters in developing nerve cells in mice during their growth and development.

## Conclusion

The results of the study suggest that the use of the psychobiotics *Lactobacillus rhamnosus* JB-1 and *Bifidobacterium longum* Rosell®-175 as diet supplements in mice enhances expression of proteins involved in the activation and maturation of nerve cells, as well as myelination and homeostatic regulation of neurogenesis. An increase in the expression of these proteins plays an important role in processes associated with neurodevelopmental, psychoactive, anxiety and depressive disorders and can be exploited in therapeutic strategies to prevent a reduction in cognitive functions. The results also indicate that the psychobiotics tested cause a decrease in the expression of proteins associated with CNS development and in synaptic transmission, thereby reducing the capacity for communication between nerve cells. Practical application of the research will require a better understanding of the mechanisms underlying the effects of psychobiotic microbes and their metabolites on neurons and neuroimmunomodulation processes. The results of the study indicate that psychobiotic bacteria can be useful in the development of biotherapeutics which could be used in auxiliary treatment of neurological disorders.

## Data Availability

All data generated during the current study are included in this article and are available from the first author.
